# Novel and atypical pathways for serotonin signaling

**DOI:** 10.12703/r/10-52

**Published:** 2021-06-01

**Authors:** Joël Bockaert, Carine Bécamel, Séverine Chaumont-Dubel, Sylvie Claeysen, Franck Vandermoere, Philippe Marin

**Affiliations:** 1 The Institute of Functional Genomics (IGF), University of Montpellier, CNRS, INSERM, Montpellier, France

**Keywords:** Serotonin, serotonylation, receptor, GPCR interacting protein, heteromerization

## Abstract

Serotonin (5-HT) appeared billions of years before 5-HT receptors and synapses. It is thus not surprising that 5-HT can control biological processes independently of its receptors. One example is serotonylation, which consists of covalent binding of 5-HT to the primary amine of glutamine. Over the past 20 years, serotonylation has been involved in the regulation of many signaling mechanisms. One of the most striking examples is the recent evidence that serotonylation of histone H3 constitutes an epigenetic mark. However, the pathophysiological role of histone H3 serotonylation remains to be discovered. All but one of the 5-HT receptors are G-protein-coupled receptors (GPCRs). The signaling pathways they control are finely tuned, and new, unexpected regulatory mechanisms are being uncovered continuously. Some 5-HT receptors (5-HT_2C_, 5-HT_4_, 5-HT_6_, and 5-HT_7_) signal through mechanisms that require neither G-proteins nor β-arrestins, the two classical and almost universal GPCR signal transducers. 5-HT_6_ receptors are constitutively activated via their association with intracellular GPCR-interacting proteins (GIPs), including neurofibromin 1, cyclin-dependent kinase 5 (Cdk5), and G-protein-regulated inducer of neurite outgrowth 1 (GPRIN1). Interactions of 5-HT_6_ receptor with Cdk5 and GPRIN1 are not concomitant but occur sequentially and play a key role in dendritic tree morphogenesis. Furthermore, 5-HT_6_ receptor-mediated G-protein signaling in neurons is different in the cell body and primary cilium, where it is modulated by smoothened receptor activation. Finally, 5-HT_2A_ receptors form heteromers with mGlu_2_ metabotropic glutamate receptors. This heteromerization results in a specific phosphorylation of mGlu_2_ receptor on a serine residue (Ser^843^) upon agonist stimulation of 5-HT_2A_ or mGlu_2_ receptor. mGlu_2_ receptor phosphorylation on Ser^843^ is an essential step in engagement of G_i/o_ signaling not only upon mGlu_2_ receptor activation but also following 5-HT_2A_ receptor activation, and thus represents a key molecular event underlying functional crosstalk between both receptors.

## Introduction

**Figure 1.**
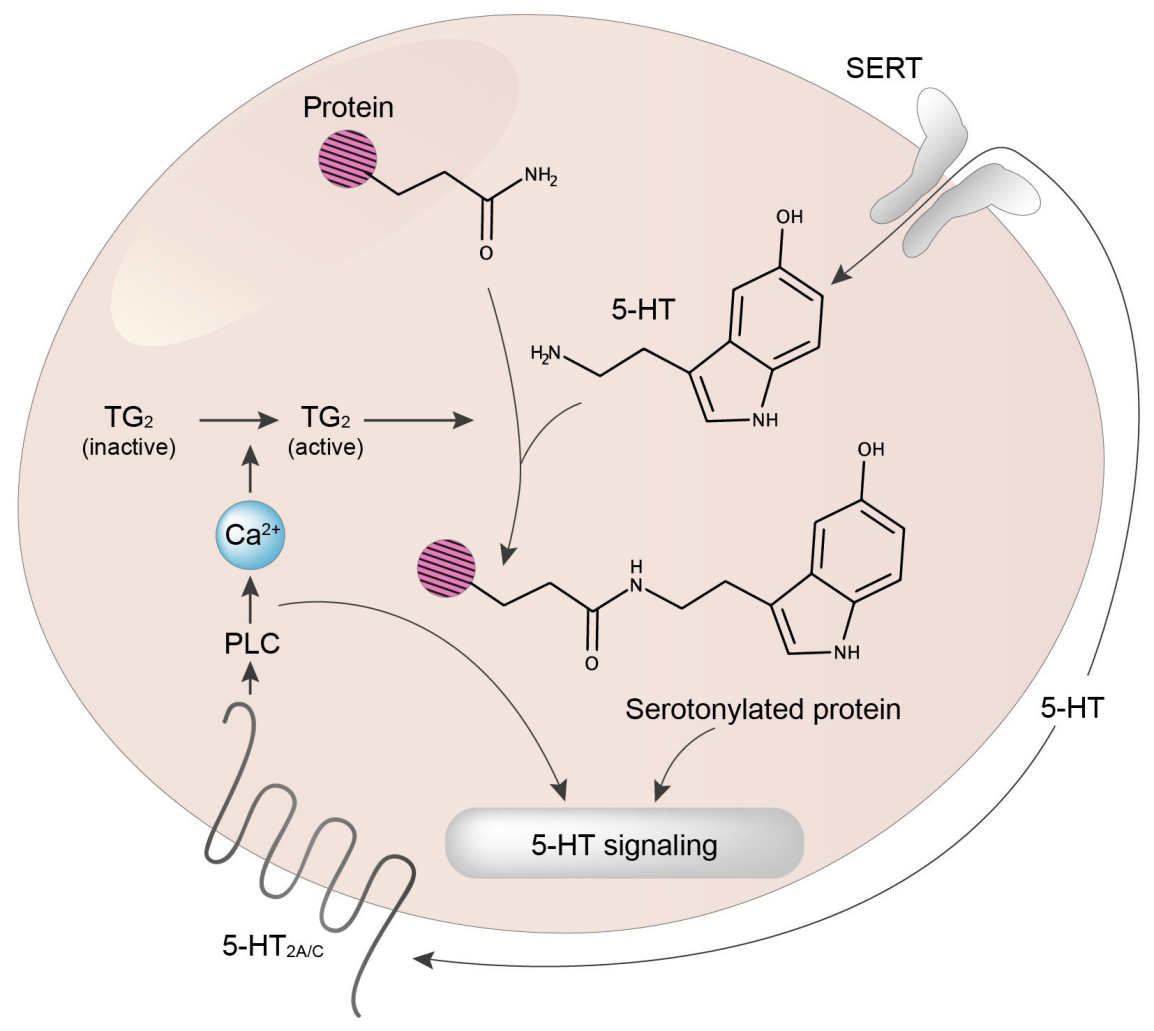
5-HT signaling via G-protein-coupled receptors (GPCRs) and serotonylation. 5-HT controls cell signaling via its cognate receptors (mostly GPCRs) and intracellularly via transamidation (serotonylation) of specific proteins. Note that extracellular proteins can also be serotonylated (not illustrated). Owing to the hydrophilic nature of 5-HT, serotonylation is believed to occur only in cells expressing the serotonin transporter (SERT). In the example illustrated in the figure, 5-HT_2A/C_ receptor stimulation by 5-HT induces activation of phospholipase C (PLC), and thereby an increase in intracellular Ca^2+^ concentration, a process leading to a full activation of the transglutaminase TG2.

The serotonin (5-HT) biosynthetic pathway is an ancestral biological process present in unicellular systems such as cyanobacteria, green algae, and fungi and is conserved in both invertebrates and vertebrates^1^. In contrast, 5-HT receptors have not been found in plants and appeared along with synapses 600 million years ago^1^. Therefore, it is not surprising that some 5-HT biological effects that do not require 5-HT receptors have been established during evolution. One of the most fascinating discoveries of the last 20 years is the demonstration that 5-HT can bind covalently to the primary amine of glutamine in proteins^2,3^. This covalent modification called serotonylation is implicated in many biological mechanisms, such as epigenetics, both at the periphery and in the brain^3–7^ Thus, 5-HT controls cellular signaling events by acting either extracellularly via membrane receptors or intracellularly via serotonylation, even though some serotonylation events occur extracellularly (Figure 1). This dual 5-HT control of cell signaling is shared with dopamine, histamine, and noradrenalin^6,7^. It illustrates the proposal from François Jacob that evolution tinkers with a limited number of disposable genes and molecules to ensure the greatest number of biological functions^8,9^.

5-HT finely controls an increasing number of functions including highly complex processes, such as anxiety, mood, learning, memory, cognition, social interactions, sleep, and appetite, but also more unexpected ones, such as shell formation in bivalves^10^. This large diversity of 5-HT functions has been made possible by selecting a large number of receptors that finely regulate diverse cellular signaling pathways, and 5-HT is certainly one of the neurotransmitters able to activate the largest number of receptor subtypes (17 identified in vertebrates). All are G-protein-coupled receptors (GPCRs), except for the five 5-HT_3_ receptors, which are cation channels^11^. Twenty years ago, the signaling mechanisms associated with 5-HT GPCRs were thought to be simple^12^. The 5-HT_1_ receptor family was known to be coupled to G_i_ proteins, thus inhibiting adenylyl cyclase (AC), the 5-HT_2_ family to Gq (activate phospholipase C), and 5-HT_4_, 5-HT_6_, and 5-HT_7_ receptors to Gs (activate AC), while the coupling mechanisms of 5-HT_5_ receptors remained elusive. Our current knowledge of 5-HT receptor signal transduction is now much more complex than this initial view and is in constant evolution^11,13^. Since 5-HT receptor-mediated signaling has been extensively reviewed elsewhere^11,13–17^, we will focus here on the most original and intriguing signaling mechanisms that have been recently described.

## Receptor-independent 5-HT signaling: serotonylation

The covalent binding of polyamines or biogenic monoamines (serotonin, dopamine, noradrenalin) to glutamine was described a long time ago^3^. Enzymes responsible for this biochemical reaction, called transamidation, are transglutaminases (TGs)^3^. Seven TGs exhibiting mainly intracellular location have been identified, the most abundant and ubiquitous one being TG2. Blood coagulation factor XIII, once activated by thrombin during coagulation to give factor XIIIa, also displays extracellular TG activity^3^.

**Figure 2.**
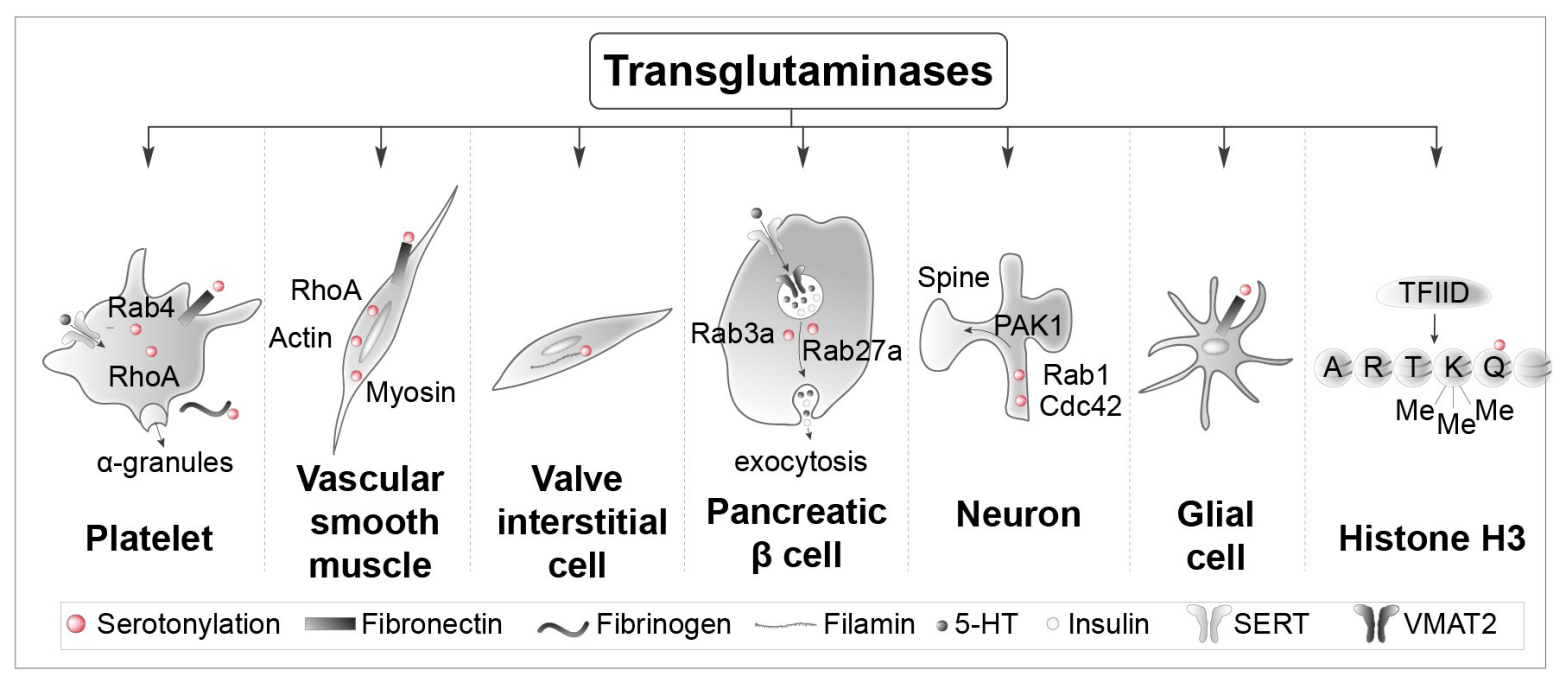
Specific proteins serotonylated by transglutaminases in different cell types. Examples of intracellular and extracellular serotonylated proteins in platelets, vascular smooth muscle cells, valve interstitial cells, pancreatic β-cells, neurons, and glial cells are illustrated. The right panel shows serotonylated histone H3 at position 5 (Q5ser) by transglutaminase 2 (TG2) predominantly in combination with trimethylation of adjacent lysine (K)4, resulting in the double epigenetic mark H3K4me3Q5ser. SERT, serotonin transporter; TFIID, transcription factor II D; VMAT2, vesicular monoamine transporter 2.

The first physiological function depending on serotonylation was described by Dale *et al.* in 2002–2003^3,5^. This group of investigators showed serotonylation by factor XIIIa of several procoagulant proteins, including fibrinogen, von Willebrand factor, fibronectin, factor V, and thrombospondin on the surface of activated platelets, which leads to the accumulation of aggregated proteins in the extracellular matrix^18^. Fibrin is also cross-linked by factor XIII, thereby increasing clot resistance^19^. Following the observation that *Bordetella pertussis* toxin acts as a transglutaminase that covalently binds polyamines to small G-proteins^20^, Walther *et al.* discovered that small G-proteins (RhoA and Rab4) are serotonylated (likely by TG2) in platelets, a process making them constitutively active in a GTP-bound form^2^ (Figure 2). RhoA reorganizes the cytoskeleton, whereas Rab4 stimulates the exocytosis of α-granules, which contain proteins involved in coagulation. A rise in intracellular Ca^2+^ is necessary to activate TG2^2^. This Ca^2+^ elevation is due, at least in part, to the activation of platelet 5-HT_2A_ receptors^21^. Thus, 5-HT acts both extracellularly and intracellularly during platelet activation and the serotonin transporter (SERT) is needed for intracellular accumulation of 5-HT required for serotonylation^5^.

Pulmonary hypertension involves proliferation and contraction of arterial smooth muscles. Proliferation of arterial smooth muscles is under the control of serotonylated RhoA^22,23^, whereas their contraction is modulated by serotonylated actin and myosin^24^ (Figure 2). Fibronectin serotonylation has also been involved in pulmonary hypertension^3^, a process favored by the up-regulation of TG2^25^. The remodeling of cardiac valve interstitial cells, a heterogeneous population of cells responsible for maintaining the structural integrity and normal functioning of the valve, is key to understanding mitral and aortic valve dysfunctions in pulmonary hypertension. The role of 5-HT_2B_ receptor activation by 5-HT in this pathology caused by fenfluramine treatment is well known, but serotonylation of filamin-A has also been implicated^26^ (Figure 2). Pancreatic β-cells capture extracellular 5-HT via SERT. Cytosolic 5-HT in turn accumulates with insulin in secretory vesicles through vesicular monoamine transporter 2 (VMAT2). Exocytosis of vesicles and co-release of 5-HT and insulin also require serotonylation of two small G-proteins, Rab3a and Rab27a^27^ (Figure 2). Stimulation of 5-HT_2A/2C_ receptors by 1-[2,5-dimethoxy-4-iodophenyl]-2-aminopropane (DOI) induces Ca^2+^-dependent TG activation, serotonylation of Rac1 and Cdc42, and Pak1 stimulation in cortical neurons, a process leading to an increase in dendritic spine size (Figure 2)^28^. This identifies serotonylation as a novel signaling pathway underlying the influence of 5-HT on dendritic spine morphology and plasticity^28^. Fibronectin and other proteins are likewise transamidated by biogenic amines including 5-HT in glial cells (Figure 2), but few data are published on the role of this chemical modification in this cell type^29^.

The most exciting discovery on transamidation is its role as an epigenetic mark controlling gene expression^6,7^. Serotonylation of histone H3 on glutamine (Q5) has been found in several organs producing 5-HT, including the brain and the gut, and in different animal species^4^ (Figure 2). 5-HT and other monoamines are present in the nucleus^30,31^ and can thus serve as substrates for transamidation of histones. The nuclear membrane is permeable to monoamines^32^, which allows a rapid equilibration of extravesicular monoamines between the cytoplasm and the nucleus^30^. Nuclear 5-HT is mobilized and released upon stimulation of dorsal raphe-containing brain slices^30^.

Histone H3 serotonylation requires trimethylation of the neighboring lysine (K4) and occurs during the differentiation of human pluripotent stem cells into 5-HT-containing neurons. This histone H3 modification is enriched in gene promoters and facilitates binding of the general transcription factor TFIID and gene transcription^4^. Similarly, dopaminylation of Q5 of histone H3 was found during cocaine withdrawal. This promotes gene expression in ventral tegmental area (VTA) neurons, increases their excitability, and favors drug-seeking behavior in rats^6,7^.

## G-protein-independent signaling at 5-HT G-protein-coupled receptors

Like many proteins, GPCRs can adopt different active and inactive conformations^33–35^. Some active conformations favor activation of one or several G-proteins, whereas others favor GPCR association with β-arrestins^35^. Biased ligands can stabilize either one or several G-protein-preferring conformations, or β-arrestin-preferring conformations, or conformations favoring both G-protein- and β-arrestin-dependent signaling. In addition, some GPCRs, including 5-HT receptors, trigger signaling events without any involvement of G-proteins or β-arrestins, that can thus be designated as “non-G-protein/β-arrestin” signaling. These particular signaling mechanisms can result from either receptor stabilization in a specific active conformation by binding to an agonist or, in some cases, agonist-independent (constitutive) receptor activation.

**Figure 3.**
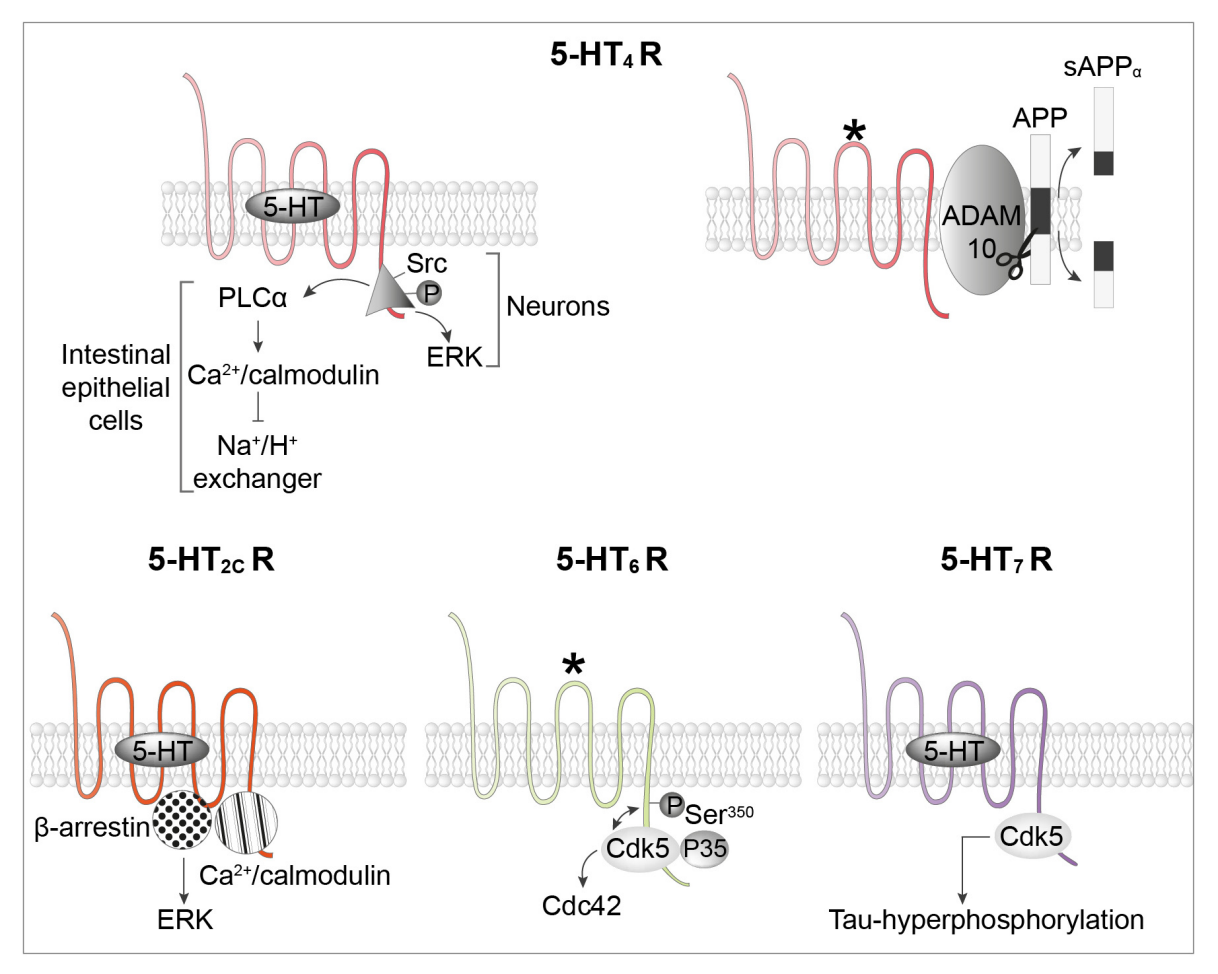
Non-G-protein signaling at 5-HT receptors. Top: the 5-HT_4_ receptor engages Erk1,2 signaling in neurons through a Gs- and β-arrestin-independent mechanism that requires activation of the non-receptor tyrosine kinase Src. In the intestinal epithelial Caco-2 cell line, 5-HT_4_ receptor-mediated Src activation leads to phospholipase C (PLC)/Ca^2+^-calmodulin-dependent inhibition of the Na^+^/H^+^ exchanger. *Constitutively active receptors. Bottom left: the 5-HT_2C_ receptor engages Erk1,2 signaling in neurons through a G-protein-independent, β-arrestin-dependent mechanism that requires physical association of calmodulin with the receptor’s C-terminal domain. Bottom, middle: the 5-HT_6_ receptor activates the cyclin-dependent kinase 5 (Cdk5)–Cdc42 signaling pathway in an agonist-independent manner through a reciprocal interplay between the receptor and associated Cdk5, by which Cdk5 bound to the receptor C-terminal domain phosphorylates the receptor on Ser^350^, a necessary step in Cdk5-dependent activation of Cdc42. Bottom, right: the 5-HT_7_ receptor binds to and activates Cdk5 signaling, a process leading to Tau hyperphosphorylation. APP, amyloid precursor protein.

While many GPCRs are known to stimulate the MAP-kinase Erk1,2 pathway through the sequential activation of G-proteins and β-arrestins^36^, two 5-HT receptor subtypes have been shown to engage the Erk1,2 signaling pathway through G-protein- or β-arrestin-independent mechanisms. Stimulation of Erk1,2 by the 5-HT_2C_ receptor does not require G-proteins and entirely depends on the concomitant recruitment of β-arrestin and calmodulin (CaM) by the receptor^37^. In light of the direct interaction of purified calmodulin (CaM, bound to Ca^2+^) with β-arrestin^38^ and CaM dimerization^39^, it has been proposed that β-arrestin might be recruited via CaM bound to the receptor, which might also stabilize the 5-HT_2C_ receptor/β-arrestin complex (Figure 3). Consistent with this hypothesis, Erk1,2 activation by the 5-HT_2C_ receptor is unusually long lasting (up to 3 hours) when compared to other GPCRs^37^. This contrasts with the activation of Erk1,2 signaling by the 5-HT_4_ receptor, which lasts only 20 minutes and is independent of both G-proteins and β-arrestins but requires Src activation (Figure 3)^40^. In the Caco-2 epithelial intestinal cell line, 5-HT_4_ receptor-mediated Src activation also leads to PLC/Ca^2+^-CaM-dependent inhibition of the Na^+^/H^+^ exchanger^41^.

The 5-HT_4_ receptor displays a high level of constitutive activity. Constitutively active 5-HT_4_ receptors directly bind to the α-secretase ADAM10 and stimulate its activity^42^, thus favoring non-amyloidogenic cleavage of the amyloid precursor protein (APP, Figure 3). Stimulation of ADAM10 by constitutively active 5-HT_4_ receptors is independent of Gs and cAMP production^42^. The conformation of constitutively active 5-HT_4_ receptors associated with ADAM10 likely differs from the conformation of constitutively active 5-HT_4_ receptors coupled to Gs. Indeed, whereas agonist-independent 5-HT_4_ receptor-operated Gs signaling is inhibited by the inverse agonists RO 116-0086 and RO 116-2617, these compounds are inactive on receptor-dependent ADAM10 activation^42^. Chronic administration of RS 67333, a 5-HT_4_ receptor agonist, or donecopride, a multi-target compound able to both inhibit acetylcholinesterase and activate 5-HT_4_ receptors, decreases amyloid load and Tau hyperphosphorylation as well as learning and memory deficits in mouse models of Alzheimer's disease^43,44^.

Native 5-HT_6_ receptors exhibit a high level of constitutive activity at Gs signaling. The 5-HT_6_ receptor also constitutively activates cyclin-dependent kinase 5 (Cdk5)/Cdc42 signaling through a mechanism involving agonist-independent association of Cdk5 and its activator p35 to the receptor C-terminus and receptor phosphorylation on a Ser residue (Ser^350^) by associated Cdk5^45^ (Figure 3). This pathway is engaged by mutated 5-HT_6_ receptors unable to activate Gs, suggesting the presence of at least two different active receptor conformations able to activate Gs and Cdk5 signaling, respectively^14,45^. Agonist-independent 5-HT_6_ receptor-operated Cdk5 signaling finely tunes cortical neuron migration and promotes the initiation of neurite growth^45–47^.

Likewise, constitutively active 5-HT_7_ receptors directly bind to and activate Cdk5 in a G-protein-independent manner (Figure 3)^48^. In a mouse model of tauopathy overexpressing a human Tau mutant known to be associated with frontotemporal dementia (R^406^W), constitutively active 5-HT_7_ receptors physically associated with Cdk5 induce hyperphosphorylation of Tau and the formation of highly bundled Tau structures^48^, suggesting that the 5-HT_7_ receptor–Cdk5 signaling pathway may be a new target in tauopathies.

## Constitutive activation of 5-HT_6_ receptor by interacting proteins

Another example of molecular tinkering of GPCRs^8^ is their ability to be activated by both agonists and their interaction with intracellular proteins. Twenty years ago, we described the first example of agonist-independent activation of GPCRs (the group I metabotropic glutamate receptors mGlu_1_ and mGlu_5_) by an intracellular GPCR-interacting protein (GIP), Homer1a. Homer1a is the product of an immediate early gene induced in activated neurons^49^. Constitutively active mGlu_1_/Homer1a and mGlu_5_/Homer1a complexes are implicated in a large series of homeostatic plasticity events^50–54^.

**Figure 4.**
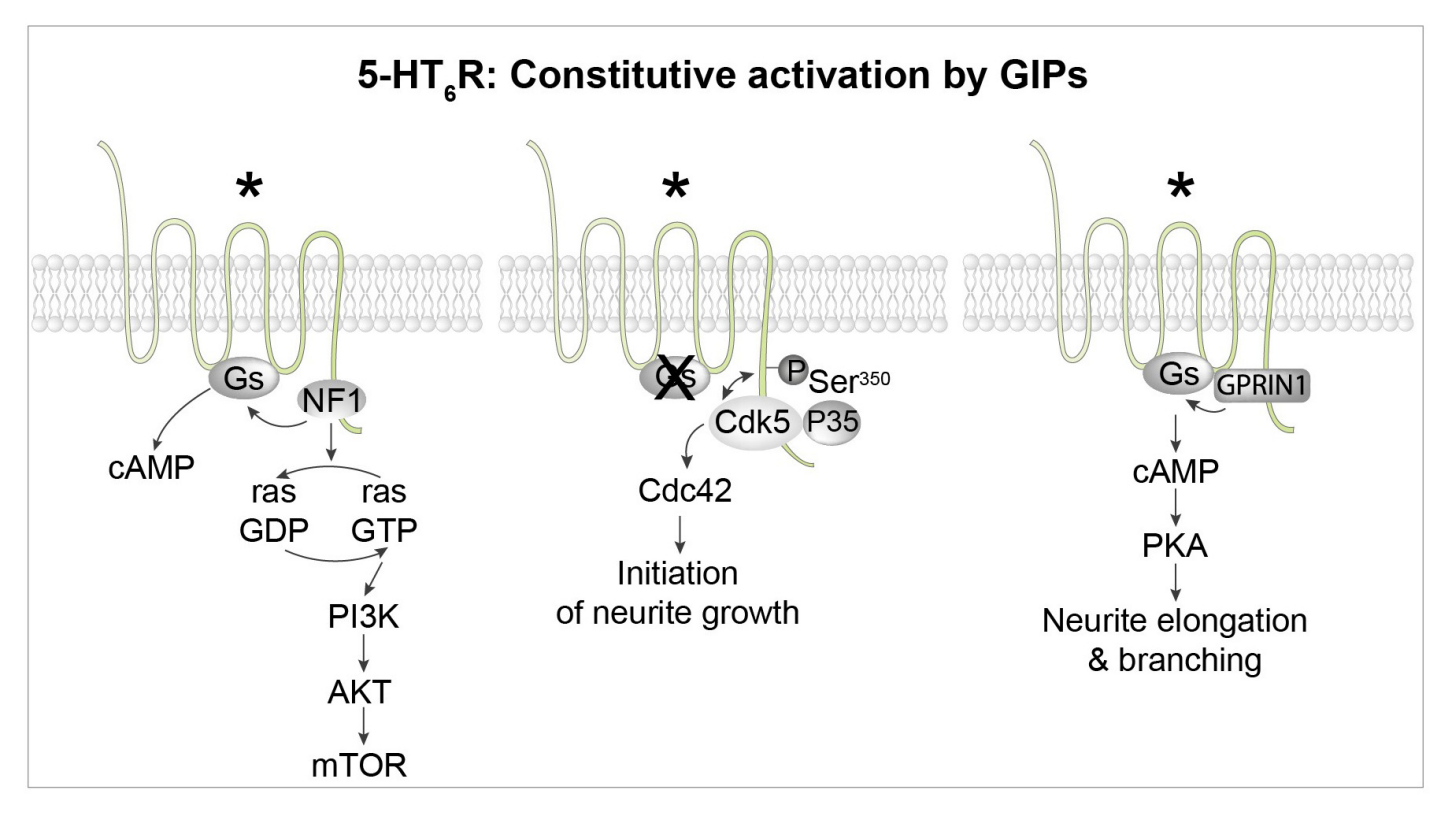
Constitutive activation of 5-HT_6_ receptors by different GPCR-interacting proteins (GIPs). Left: The 5-HT_6_ receptor binds to several proteins of the mammalian target of rapamycin complex 1 (mTORC1) pathway, including mTOR itself and the Ras GTPase-activating protein (Ras-GAP) neurofibromin 1 (NF1). Physical association of NF1 with the receptor strongly enhances constitutive activation of the Gs–adenylyl cyclase pathway by the receptor. Middle: The receptor activates the cyclin-dependent kinase 5 (Cdk5)–Cdc42 signaling pathway in an agonist-independent manner to promote the initiation of neurite growth. Dissociation of the 5-HT_6_ receptor–Cdk5 complex allows the recruitment of G-protein-regulated inducer of neurite outgrowth 1 (GPRIN1) by the receptor (right panel), which mediates constitutive activation of the Gs–adenylyl cyclase–protein kinase A (PKA) pathway, thereby promoting neurite elongation and branching. *Constitutively active receptor. PI3K, phosphatidylinositol 3-kinase; Ser, serine.

More recently, we reported the association of 5-HT_6_ receptor with many GIPs. These include proteins of the mechanistic target of rapamycin (mTOR) pathway (mTOR itself, Raptor, which together with mTOR is part of the mTOR complex 1, the Ras GTPase-activating protein [Ras-GAP] neurofibromin 1, and Vps34, a class III phosphatidylinositol 3‐kinase). Further studies revealed that mTOR activation by 5-HT_6_ receptor has a deleterious influence upon cognition in rodent models of schizophrenia and cannabis abuse during adolescence^55,56^. Three 5-HT_6_ receptor-interacting proteins were found to activate 5-HT_6_ receptors in an agonist-independent manner (Figure 4)^13^. The first one is Cdk5, as already discussed^45^. The second is neurofibromin 1, a protein encoded by the tumor suppressor gene *NF1* that directly binds to the C-terminus of 5-HT_6_ receptor^14,57^. Mutations of the *NF1* gene are responsible for neurofibromatosis type 1 (Nf1), a genetic disease characterized by skin pigmentation and benign skin tumors, low-grade tumors of the central and peripheral nervous systems, and learning and attention deficits in some patients. The binding of neurofibromin 1 to 5-HT_6_ receptors strongly enhances native 5-HT_6_ receptor constitutive activity at Gs signaling (Figure 4). Correspondingly, SB 271046, a 5-H_6_ receptor inverse agonist, decreases cAMP level and downstream signaling in wild-type mice but not Nf1^+/−^ mice^57^. Likewise, blocking 5-HT_6_ receptor/neurofibromin 1 interaction by an interfering peptide strongly reduces 5-HT_6_ receptor constitutive activity in primary neurons. These findings demonstrate that physical interaction between neurofibromin 1 and 5-HT_6_ receptor enhances constitutive receptor coupling to Gs^57^. The third 5-HT_6_ receptor-interacting protein that was found to promote agonist-independent activation of Gs and cAMP production without altering the agonist-dependent response is G-protein-regulated inducer of neurite outgrowth 1 (GPRIN1)^58^ (Figure 4). The 5-HT_6 _receptor–GPRIN1 complex promotes neurite extension and branching in NG108-15 cells and mouse primary neurons through a cAMP- and PKA-dependent mechanism^58^.

Agonist-independent activation of GPCRs by GIPs generally induces more prolonged receptor activation than that elicited by classical agonists. In fact, activation of a GPCR by a GIP will last as long as the protein is bound to the receptor. Accordingly, GIP-dependent GPCR activation can last hours (as shown for mGlu1/5 receptor activation by Homer1a) or even be “permanent”, such as 5-HT_6_ receptor constitutive activation upon association with neurofibromin 1. The reversibility of receptor activation will depend only on GIP protein turnover.

## Biased agonism at 5-HT_2A_ receptor: impact of its heteromerization with mGlu_2_ receptor

As already discussed, GPCRs transduce signal not only via the activation of one or several G-proteins or its binding to β-arrestins but also via non-G-protein/non-β-arrestin pathways. Different ligands of a given GPCR can preferentially stimulate either G-protein- or β-arrestin-dependent signaling, a phenomenon known as biased signaling^59^. The extreme situation is a ligand displaying no efficacy in promoting receptor coupling to G-proteins but serving as an agonist for β-arrestin-mediated signaling^59^. This phenomenon has been called “biased agonism” or “functional selectivity”^60^. Consequently, depending on the pattern of signaling pathways selected by a given GPCR ligand, cellular and physiological responses will differ. Functional selectivity raised great interest in the pharmaceutical industry with the perspective of developing drugs able to activate signaling pathways underlying therapeutic response but not those responsible for side effects^61^.

**Figure 5.**
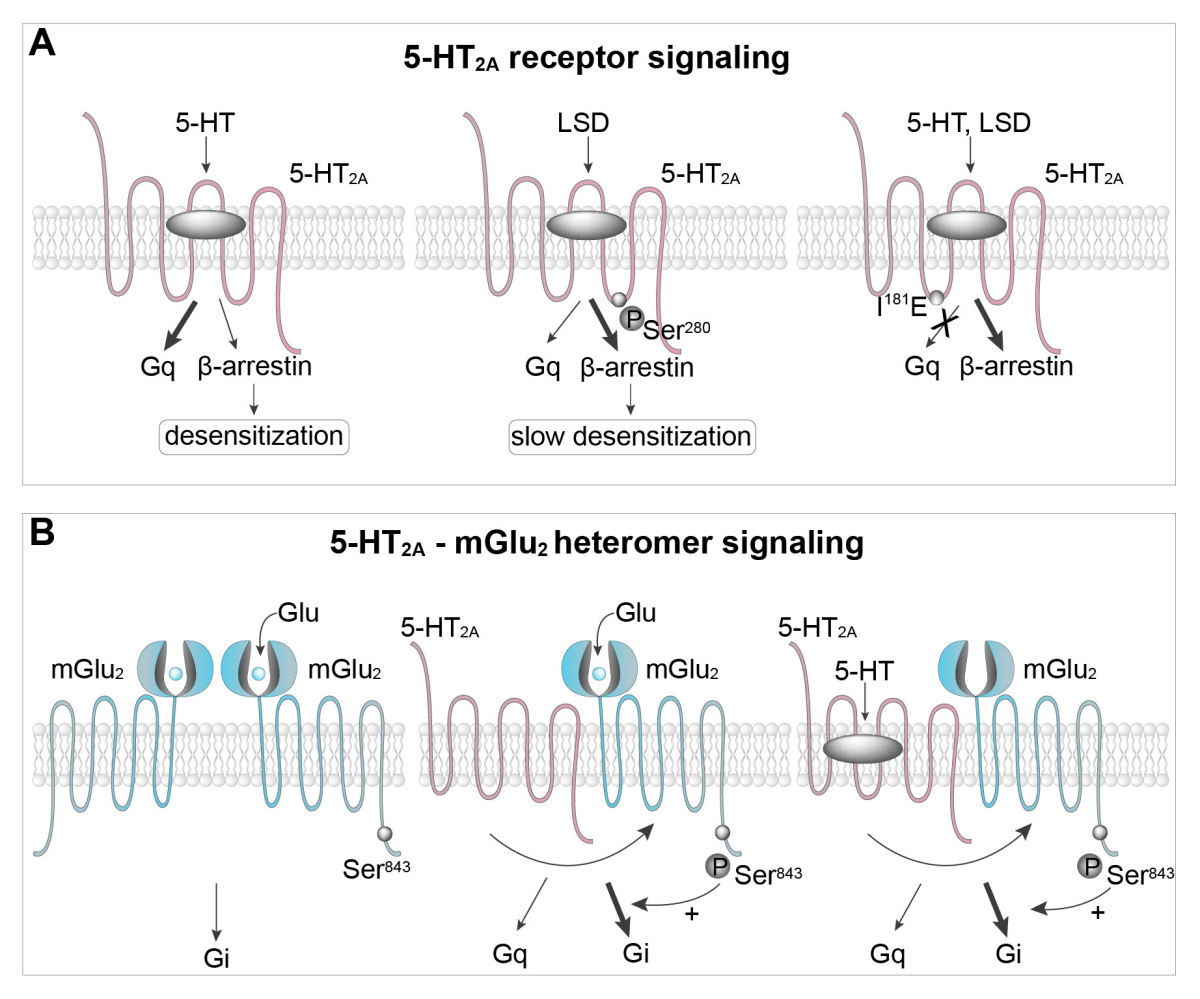
Biased signaling at 5-HT_2A_ receptors and 5-HT_2A_/mGlu_2_ heteromers. **A.** 5-HT_2A_ receptor stimulation by 5-HT activates Gα_q_ and, to a lesser extent, β-arrestin signalling and leads to 5-HT_2A_ receptor desensitization. 5-HT_2A_ receptor stimulation by psychedelic hallucinogens such as lysergic acid diethylamide (LSD), but not by non-hallucinogenic agonists, promotes receptor phosphorylation on serine (Ser)^280^, a process reducing receptor desensitization. Binding of the receptor to hallucinogenic agonists also stabilizes conformations favoring β-arrestin coupling^66^. Substituting isoleucine^181^ of the receptor to glutamate suppresses receptor coupling to Gα_q_ while potentiating coupling to β-arrestin upon receptor activation by 5-HT or hallucinogenic agonists. **B.** Agonist stimulation of metabotropic glutamate receptor 2 (mGlu_2_) or 5-HT_2A_ receptor within mGlu_2_–5-HT_2A_ heterodimer promotes mGlu_2_ receptor phosphorylation on Ser^843^, which favors engagement of Gα_i_ signaling.

As for many GPCRs, biased ligands acting on 5-HT receptors are actively searched in order to obtain more selective drugs with fewer side effects^62–65^. Agonists acting at 5-HT_2A_ receptors represent one of the most striking illustrations of functional selectivity^66,67^. Some 5-HT_2A_ receptor agonists like lysergic acid diethylamide (LSD), psilocybin (“magic mushrooms” drug), or DOI (“designer drug”) trigger hallucinations, whereas its natural ligand 5-HT and other agonists like the anti-parkinsonian compound lisuride or the anti-migraine drug ergotamine do not trigger such psychoactive effects. 5-HT_2A_ receptors are canonically coupled to both the Gα_q_ protein family and β-arrestin and quickly desensitized upon 5-HT stimulation^11^. LSD is a β-arrestin-biased ligand that promotes preferential 5-HT_2A_ receptor coupling to β-arrestin compared with 5-HT^67^ (Figure 5). Consistent with these findings, structural studies indicate that the conformation adopted by the structurally related 5-HT_2B_ receptor bound to LSD slightly differs from the conformation elicited by non-hallucinogenic agonists. The most important difference is a more constrained conformation of extracellular loop 2 near the orthosteric site, which causes a more prolonged residence time of LSD, leading to a stronger and more prolonged β-arrestin recruitment^68^. Recently, a high-resolution structure of hallucinogen-bound 5-HT_2A_ receptor also revealed how hallucinogens stabilize states favoring β-arrestin coupling^67^. Interestingly, substituting a hydrophobic residue within the intracellular loop 2, essential for coupling of various GPCRs to G-proteins (isoleucine^181^in 5-HT_2A_ receptor) into glutamate, suppresses receptor coupling to Gα_q_ while potentiating coupling to β-arrestin^67^ (Figure 5).

The comparison of phosphoproteomes in 5-HT_2A_ receptor-expressing recombinant cells challenged with either the hallucinogenic agonist DOI or the non-hallucinogenic agonist lisuride revealed that among thousands of quantified phosphorylated residues, only a few of them are specifically phosphorylated upon exposure to DOI, but not lisuride. These include a serine residue (Ser^280^) located in the third intracellular loop of the 5-HT_2A_ receptor itself. The specific phosphorylation of Ser^280^ upon 5-HT_2A_ receptor stimulation by hallucinogenic agonists was then established *in vivo*, in mouse prefrontal cortex^69^ (Figure 5). Further functional studies revealed that this biased phosphorylation event is responsible for a reduced desensitization of 5-HT_2A_ receptor when stimulated by hallucinogenic *vs.* non-hallucinogenic agonists^69^. This attenuated 5-HT_2A_ receptor desensitization following stimulation by hallucinogenic agonists results in more sustained receptor activation that might contribute, at least in part, to their psychotropic effects.

The ability of the 5-HT_2A_ receptor to couple to Gα_i_ and Gα_s_ proteins in addition to Gα_q_ is still controversial^67,70^. An extended analysis by Kim *et al.* clearly establishes that the 5-HT_2A_ receptor is mostly coupled to the Gq protein family upon stimulation by either 5-HT or LSD^67^. 5-HT_2A_ receptors can form heteromers with the mGlu_2_ receptor, a Gα_i_ protein-coupled receptor^71^ (Figure 5). Within the heteromer, the respective coupling of each protomer to its cognate G-protein is oppositely influenced by the other protomer: while 5-HT_2A_ receptor coupling to Gα_q_ in response to agonist stimulation is decreased by approximately 50% within heteromers, compared with 5-HT_2A_ receptor not associated with mGlu_2_ receptor, Gα_i_ activation elicited by agonist stimulation of the mGlu_2_ receptor is strongly potentiated by its heteromerization with the 5-HT_2A_ receptor^72^. A recent study revealed that the 5-HT_2A_ receptor also affects mGlu_2_ receptor trafficking and subcellular localization through a mechanism dependent on their heterodimerization^73^.

We demonstrated that 5-HT_2A_ receptor co-expression is required for the phosphorylation of the mGlu_2_ receptor on a serine located in its C-terminal domain (Ser^843^) upon mGlu_2_ receptor stimulation by the orthosteric agonist LY379268 in recombinant cells^74^. Furthermore, phosphorylation of Ser^843^ elicited by mGlu_2_ receptor stimulation is blocked by a 5-HT_2A_ receptor antagonist (Figure 5). Corroborating these observations in cell cultures, *in vivo* administration of LY379268 increases mGlu_2_ receptor phosphorylation at Ser^843^ in prefrontal cortex of wild-type mice but not 5-HT_2A_^−/−^ mice. Stimulation of the 5-HT_2A_ receptor also increases phosphorylation of Ser^843^, an effect blocked by mGlu_2_ receptor antagonist, thus highlighting a sophisticated crosstalk between both receptors to promote mGlu_2_ Ser^843^ phosphorylation (Figure 5). Mutation of Ser^843^ into alanine strongly reduces Gα_i/o_ signaling elicited by mGlu_2_ or 5-HT_2A_ receptor stimulation in cells co-expressing both receptors^74^. This identifies mGlu_2_ Ser^843^ phosphorylation as a mechanism by which the 5-HT_2A_ receptor can “hijack” Gα_i_ signaling within 5-HT_2A_–mGlu_2_ heteromers. It has been proposed that the balance of Gα_q_ *vs.* Gα_i_ signaling at 5-HT_2A_–mGlu_2_ heteromers determines pro-psychotic *vs.* antipsychotic activity of ligands of each of these receptors^72^. Given the critical influence of Ser^843^ phosphorylation on Gα_i/o_ signaling at 5-HT_2A_–mGlu_2_ heteromers, alterations of its phosphorylation level might be a key event underlying the pathogenesis of psychotic disorders such as schizophrenia as well as the behavioral effects of psychedelic drugs and antipsychotics.

## 5-HT receptor spatiotemporal signaling

GPCR signal transduction is not stable over time, even in the presence of constant agonist stimulation. In addition, it depends on receptor subcellular localization. Most GPCRs undergo desensitization upon agonist stimulation, leading to a decline of G-protein-dependent signaling while alternative pathways, such as the β-arrestin-dependent pathway, are enhanced^9,75,76^. Many GPCRs are internalized in endosomes upon prolonged agonist stimulation. Surprisingly, some GPCRs continue to transduce signal in endosomes, not only via β-arrestin but also, in some cases, via G-proteins^76,77^. For instance, parathormone (PTH) receptors still activate G_s_ and cAMP production in endosomes^76^.

**Figure 6.**
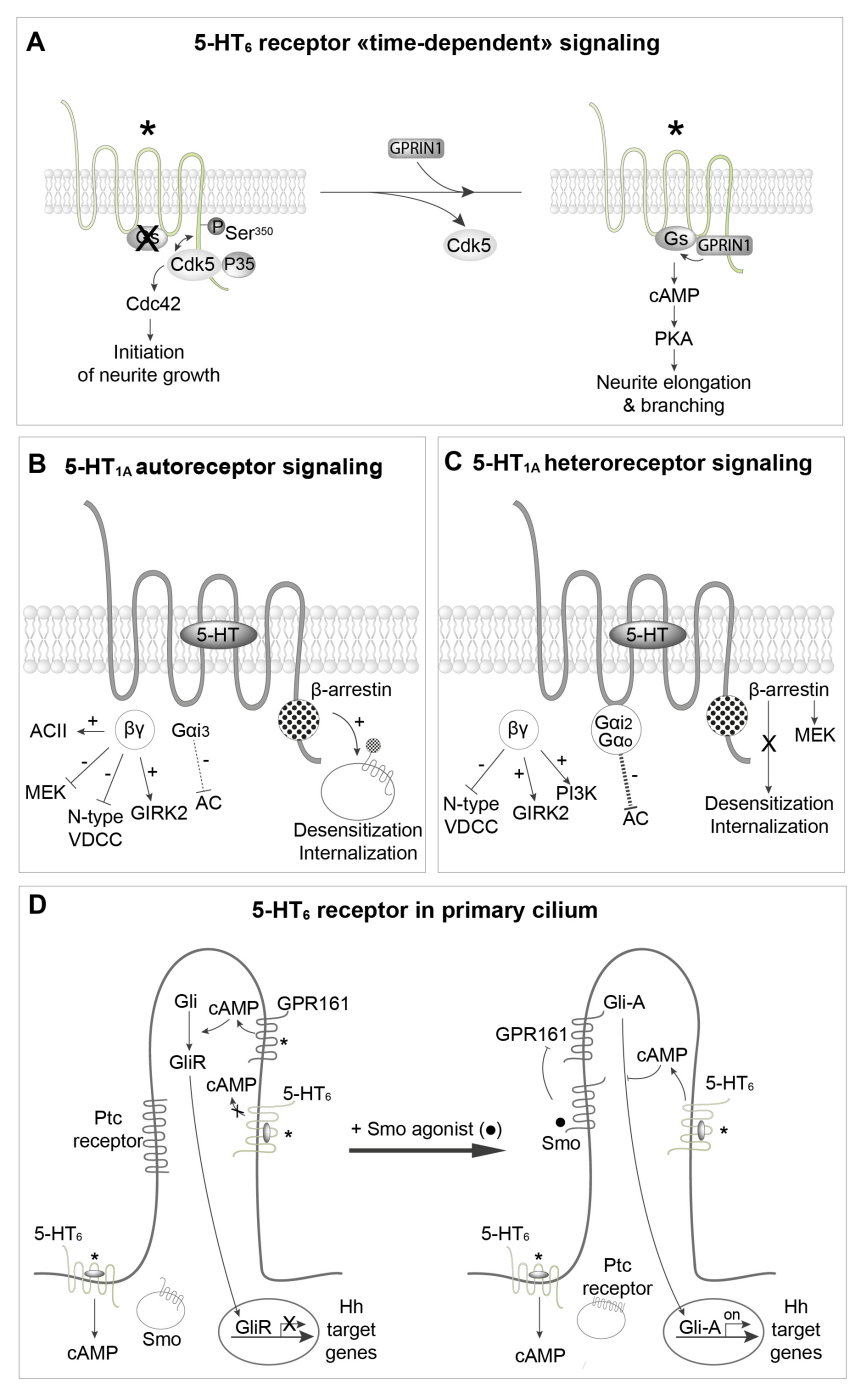
Spatiotemporal regulation of 5-HT receptor signaling. **A**. Sequential engagement cyclin-dependent kinase 5 (Cdk5)–Cdc42 and Gs–adenylyl cyclase pathways by constitutively active 5-HT_6_ receptors during neuronal differentiation and dendritic tree morphogenesis. **B**,**C**. Difference in coupling properties of 5-HT_1A_ autoreceptor and heteroreceptors and in their propensity to desensitize and internalize upon agonist stimulation. **D**. Spatiotemporal regulation of 5-HT_6_ receptor coupling to Gs in neurons. 5-HT_6_ receptors located in the soma, but not receptors located in the primary cilium, activate cAMP production (in absence or presence of agonist). Upon agonist stimulation, Smoothened (Smo) enters the cilium and inhibits cAMP production by constitutively active GPR161. This favors translocation of active Gli transcription factor (Gli-A) to the nucleus and the transcription of Hedgehog (Hh)-regulated genes. Concomitantly, 5-HT_6_ receptors located in the primary cilium become able to activate local cAMP production, which might exert a feedback inhibition of Gli-A. *Constitutively active receptors. AC, adenylyl cyclase; GIRK2, G-protein-gated inwardly rectifying potassium channel 2; GPRIN1, G-protein-regulated inducer of neurite outgrowth 1; PI3K, phosphatidylinositol 3-kinase; PKA, protein kinase A; Ptc, patched; Ser, serine; VDCC, voltage-dependent calcium channel.

We have recently described another example of time-dependent sequential coupling. As previously discussed, 5-HT_6_ receptors constitutively activate the Cdk5–Cdc42 pathway to stimulate the initiation of neurite outgrowth, and the Gs–AC pathway, via GPRIN1 physically associated with the receptor, to promote neurite extension and branching^45,58^. However, these two different signaling pathways are not concomitantly activated (Figure 6A). During early neuronal differentiation, Cdk5, but not GPRIN1, binds to the 5-HT_6_ receptor and the Cdc42 signaling pathway is switched “on”^58^ (Figure 6A). Subsequently, Cdk5 is released from 5-HT_6_ receptors, allowing recruitment of GPRIN1, activation of the Gs–AC pathway, cAMP production, and neurite extension and branching^58^ (Figure 6A).

5-HT_1A_ receptors are particularly interesting to illustrate the impact of subcellular localization of GPCRs on their signaling specificity (Figures 6B, C). 5-HT_1A_ autoreceptors are localized on 5-HT neurons on both cell bodies (in Raphe nucleus) and pre-synaptic terminals, whereas 5-HT_1A_ heteroreceptors are localized post-synaptically on target neurons^13^. It is now well established that their signaling pathways are different^13,78^. 5-HT_1A_ autoreceptors are coupled to Gα_i3_, whereas hippocampal heteroreceptors are coupled to Gα_i2_ and Gα_o_^78^. There is a consensus on the capacity of 5-HT_1A_ receptor agonists to inhibit forskolin-stimulated AC in hippocampal membranes, whereas such an inhibition seems to depend on the agonist used in Raphe nucleus membranes^78^. It is likely that the presence in the Raphe nucleus of AC type II, which is known to be stimulated by βγ released from activated Gα_i_, masks inhibition of AC by 5-HT_1A_ autoreceptors^78^.

5-HT_1A_ autoreceptors and heteroreceptors inhibit and stimulate the Erk1,2 pathway, respectively, while in the presence of mitogen FGF_2_ receptors, 5-HT_1A_ autoreceptors also stimulate the Erk1,2 pathway (Figures 6B, C). Autoreceptors and heteroreceptors also differ in their ability to desensitize. Administration of the 5-HT_1A_ receptor agonist 8-OH DPAT as well as prolonged stimulation (10–15 days) of 5-HT_1A_ autoreceptors elicited by fluoxetine or other specific serotonin reuptake inhibitors (SSRIs) result in desensitization and downregulation of 5-HT_1A_ autoreceptors, whereas heteroreceptors do not desensitize^78,79^. The reason for this difference is unknown. 5-HT_1A_ autoreceptors inhibit presynaptic 5-HT release. Since the kinetics of autoreceptor desensitization, observed following the administration of SSRIs is similar to that of their antidepressant effects, it has been suggested that desensitization of autoreceptors is mandatory to have sufficient 5-HT within the synapse to fully activate heteroreceptors^13^. Other fine differences between 5-HT_1A_ autoreceptor and heteroreceptor signaling have been recently reviewed^78^.

The 5-HT_6_ receptor represents another intriguing example of the influence of subcellular compartmentation on 5-HT receptor signal transduction. In neurons, 5-HT_6_ receptors are mainly located in the primary cilium, but they are also present at the plasma membrane of the cell body^58^ (Figure 6D). GPRIN1, which increases 5-HT_6_ receptor coupling to G_s_, is co-localized with the receptor in the cell body but not in the primary cilium^58^. Interestingly, 5-HT_6_ receptors stimulate cAMP production in the cell body but not in the primary cilium under basal conditions^80^. The more probable explanation is that coupling of 5-HT_6_ receptors to G_s_ is inhibited in the primary cilium by either a GIP or a post-translational modification, such as phosphorylation. However, no data supporting these hypotheses are so far available. In fact, a recent study suggests that 5-HT_6_ receptor-operated Gs signaling in the primary cilium can be finely regulated by a complex sequence of events depending on other ciliary receptors. When smoothened (Smo) receptor, a GPCR central in Hedgehog (Hh) signaling thought to decrease cAMP in the primary cilium through Gα_i_, is stimulated by an agonist, it enters the cilium (Figure 6D). The Hh patched (Ptc) receptor is internalized and the constitutively active Gs-coupled receptor GPR161 is inhibited. This allows activation of the Gli transcription factor (Gli-A), which translocates into the nucleus, where it induces the transcription of Hh-regulated genes^81^. 5-HT_6_ receptors concomitantly become able to activate cAMP production upon agonist receptor stimulation or as a consequence of constitutive activity, likely because they couple to G_s_, even though this remains to be demonstrated. Though much work remains to be done to understand the mechanism involved, these findings indicate a fine temporal regulation of 5-HT_6_ receptor-operated signaling in the primary cilium. Jiang *et al.* also proposed that the local production of cAMP elicited by 5-HT_6_ receptors in the cilium exerts a local feedback inhibition of Gli-A^80^ (Figure 6D).

## Conclusions and future directions

5-HT receptor signaling is not a closed chapter of pharmacology, and several important lines of research are still very active. One of them is the relationship between 5-HT receptor 3D structure and signaling. How many activated or inactivated conformations 5-HT receptors can adopt, what structural determinants are required for their alternative coupling to G-proteins, β-arrestins, and other signal transduction molecules, how biased agonists favor some of them, and how dimerization or heterodimerization influences signaling of 5-HT receptors, as established for the 5-HT_2A_–mGlu_2_ heterocomplex, remain important open questions that certainly warrant further exploration^71,72,74^. Some clues concerning the structural determinants in the 5-HT_2A_ receptor required for hallucinogen biased actions have recently been revealed^67–69^. Likewise, characterizing the constitutively active conformations selected upon interactions of the 5-HT_6_ receptor with GIPs such as neurofibromin 1, Cdk5, and GPRIN1 might be of utmost interest given the potential of this receptor as a therapeutic target for the treatment of cognitive deficits associated with neurodevelopmental disorders and dementia^45,58^. Another important line of future research concerns the spatiotemporal regulation of signaling engaged by 5-HT receptors, such as the one found for the 5-HT_6_ receptor^80^.

Another avenue of research is serotonylation^2–4^, which has a key influence on the physiology of peripheral cells, such as platelets, pancreatic β-cells, and smooth muscle cells. Surprisingly little is known about the regulation of 5-HT neuron functional activity by this biochemical process, which has long been underestimated. It is likely that the pathophysiological influence of epigenetic mechanisms related to serotonylation will also rapidly emerge in the fields of neurology and psychiatry^6,7^.
